# Assessment of the stopping for right-turning large vehicles policy in Nanjing: Effectiveness and determinants

**DOI:** 10.1371/journal.pone.0319115

**Published:** 2025-08-26

**Authors:** Yurun Zhu

**Affiliations:** School of Transportation, Southeast University, Nanjing, China; Nanjing Forestry University, CHINA

## Abstract

This study evaluates the effectiveness of Stopping for Right-Turning Large Vehicles Policy in Nanjing, designed to mitigate accidents attributed to blind spots and delayed braking of large trucks at intersections. Using high-resolution conflict data from four signalized intersections in Jiangning District, collected via unmanned aerial vehicles (UAVs) and roadside video, the research employs K-means clustering for conflict severity classification and binomial Logit regression to identify critical determinants. Results reveal the policy exhibited limited statistical significance in reducing severe conflicts (p > 0.05). Regression analysis quantified four critical determinants: absence of motorized/non-motorized segregation (OR=1.82, + 81.6% severity odds), elevated stop-line speeds (OR=1.32, + 31.9%), failure to yield (OR=2.45, + 145%), and crossing the street within the zebra crossing (OR=0.19, −81.0%). The analysis demonstrates that infrastructural deficiencies and behavioral non-compliance outweigh the policy’s standalone impact. Based on these findings, the study proposes a holistic optimization framework integrating physical separation measures, enhanced signage, dynamic traffic signal adjustments, and data-driven enforcement strategies. Methodologically, this study innovatively combines unsupervised learning for conflict categorization, providing a scalable framework for evaluating urban traffic policies. This research underscores the necessity of multi-dimensional interventions—spanning infrastructure, enforcement, and public education—to achieve sustainable improvements in intersection safety. The findings offer actionable insights for policymakers to refine regulatory measures and enhance road safety in rapidly urbanizing environments.

## 1. Introduction

Urban road intersections are primary sites for concentrated conflicts between various transport modes. Amid increasing urban construction and transport demand, large truck usage grows rapidly; compared with small cars, large trucks exhibit shortcomings including slow braking, extensive blind zones, and elevated accident rates. Notably, right-turning large vehicles at intersections frequently run over pedestrians, cyclists, or e-bike riders, resulting in sporadic casualties [1]. According to 2021 statistics, 37 right-turn accidents involving large trucks occurred in Nanjing, resulting in 24 deaths and 15 injuries [2], constituting approximately 35% of all large-truck accidents that year. Furthermore, roughly 30% of traffic fatalities involving pedestrians or non-motorized cyclists occurred when large trucks struck them during right turns at intersections [3].

In order to prevent the intersection of large trucks turning right safety accidents, Shanghai, Shenzhen, Hangzhou, Nanjing and other cities are piloting Stopping for Right-Turning Large Vehicles Policy, the provisions of large trucks into the intersection after stopping to observe, to ensure the safety of the intersection and then drive slowly through the intersection, the offender fine of 200 yuan deduction of 3 points. Globally, similar measures, such as New York City’s Turning Traffic Must Yield to PedestriansLaw and the European Directive on Heavy Goods Vehicle Blind Spot Mitigation, have shown mixed outcomes, highlighting the complexity of balancing regulatory enforcement with behavioral and infrastructural adaptations.

Conflict severity analysis remains central to intersection safety research. Domestically, Lan et al. [4] pioneered K-means clustering for conflict quantification, while Wu [5] developed threshold models to categorize conflicts. Studies on large-truck right-turn risks have identified key mechanisms: Chu et al. [6] mathematically modeled inner wheel differential, and Xu [7] and Fang et al.[8] analyzed blind-spot formation via dynamic simulation. Internationally, interventions like physical segregation (Berlin) and intelligent speed adaptation (Tokyo) reduced conflicts by 40–60%, revealing that isolated regulatory measures require complementary solutions.

Methodologically, Logit/Probit models dominate accident severity analysis [9–13]. However, emerging techniques face limitations: machine learning (ML) and artificial neural networks (ANN) enhance prediction but fail to clarify causal mechanisms (e.g., whether safety gains stem from speed reduction or signage improvements) [14, 15]. Critically, three research gaps persist:

(1) Limited evaluation of policy effectiveness;
(2) Insufficient integration of infrastructure, behavioral, and enforcement factors;
(3) Overreliance on theoretical models without empirical validation in complex urban contexts.

This study combines K-means clustering to classify conflict severity with a binomial Logit model to identify key determinants, such as vehicle speed at stop lines, yielding behavior, and zebra crossing utilization. This study innovatively explores the impact of other factors, such as facilities for separating motor and non-motor vehicles on the entry lane, and speed at the stop line, expanding the research horizons of the factors affecting traffic safety. Through the long-term tracking and evaluation of the effects of the policy, the traffic policy can be continuously optimised and adjusted, assisting the traffic management department to achieve continuous improvement and development.

## 2. Materials and methods

### 2.1. Data sources

#### 2.1.1. Conflict data acquisition.

Conflict data between right-turning large vehicles and pedestrians/non-motorized vehicles at intersections were collected using UAVs and roadside video. Four signal-controlled intersections in Jiangning District, Nanjing, were selected according to signal settings, conflict frequency, and traffic conditions. These include Liyuan Road-Tianyuan Road, Shuanglong Avenue-Qingshuting Road, Suyuan Avenue-Chengxin Avenue, and Suyuan Avenue-Qingshuting Road intersections. Two intersections with safety measures (green barriers and danger zone signs) were the experimental group, while the other two were controls, as listed in Table 1. Moreover, these four junctions have only one right-turn-only lane in both the east-west and north- and south-bound. Data were recorded during morning and evening peaks over two days, with cameras capturing opposite directions for effective analysis.

**Table 1:** Basic information of four intersections.

Serial Number	Intersection Name	Orientations	Pedestrian Crossing Width/ m	Number of Right Turn Lanes	Turn Right Green Belt	Turn Right Hazardous Area Sign
A	Liyuan Road – Tianyuan Road	east and west	6.2	1	No	No
		north and south	5.2			
B	Suyuan Avenue – Qingshuiting Road	east and west	5.6	1	No	No
		north and south				
C	Shuanglong Avenue – Qingshuiting Road	east and west	6	1	Yes	Yes
		north and south				
D	Suyuan Avenue – Chengxin Avenue	east and west	4.9	1	Yes	Yes
		north and south	4.8			

Video data were segmented into 30-second clips showing the interaction between right-turning vehicles and pedestrians/non-motorized vehicles. Using Tracker software, positional coordinates, speed, and other key data were extracted efficiently. There are a total of 288 conflict data, of which 144 are in each of the experimental and control groups, forming the basis for subsequent analysis.

#### 2.1.2. Independent Variables.

A binary Logit regression model was used to analyze conflicts, with 18 independent variables chosen from environmental, vehicle, and pedestrian aspects. Firstly, the multivariate statistical analysis methods of variance inflation factor (VIF) analysis and correlation coefficient matrix test were applied to all the independent variables. The results show that VIF of each variable ranges from 2.51 to 5.70, and the absolute value of correlation coefficient between the variables in the correlation coefficient matrix ranges from 0.35 to 0.47. Combined with the above analysis, it can be determined that there is no multicollinearity problem between these 18 independent variables, thus providing a solid and reliable variable basis for subsequent model development in terms of stability and interpretability. Totally 18 modeling independent variables selected and their frequency counts are shown in Table 2.

**Table 2:** Binary Logit Regression Model Dependent Variable Settings and Frequencies.

Variable type	Independent variable	Retrieve a value	Frequency	Percent
Environmental factor	Carriageway	Straight right lane (0)	71	41.50%
		Right turn only (1)	100	58.50%
	Non-isolated for imported machines	None (0)	107	62.60%
		Soft isolation (1)	14	8.10%
		Hard isolation (2)	50	29.30%
	Outlet non-isolated	None (0)	110	64.30%
		Soft isolation (1)	5	2.90%
		Hard isolation (2)	56	32.80%
	Formulation	Conventional (0)	80	46.80%
		Marked (1)	91	53.20%
Motor vehicle factor	Speed - 20m			
	Speed-Stop Line			
	Racing state	First car (0)	96	56.30%
		Follower car (1)	75	43.70%
	Merge into traffic	None (0)	91	53.10%
		Yes (1)	80	46.90%
	Yield	Failure to yield (0)	45	26.10%
		Slow down and give way (1)	110	64.20%
		Stop and give way (2)	16	9.70%
Non-motorized vehicles, pedestrian factors	Typology	Pedestrian (0)	11	6.30%
		Bicycle (1)	11	6.30%
		Electric car (2)	149	87.40%
	Old man(or woman)	None (0)	168	98.40%
		Yes (1)	3	1.60%
	Child	None (0)	163	95.20%
		Yes (1)	8	4.80%
	Distinguishing between the sexes	Male (0)	118	69.10%
		Female (1)	53	30.90%
	Crossing the street within the zebra crossing	No (0)	137	80.10%
		Yes (1)	34	19.90%
	Number of stops	0	152	88.80%
		1	19	11.20%
	Avoiding action	None (0)	68	39.60%
		Deceleration (1)	75	43.60%
		Acceleration (2)	28	16.80%
	Waiting time			
	Average speed			

### 2.2. Methods

#### 2.2.1. Traffic conflict metrics.

Based on the research of scholars at home and abroad on human-vehicle conflict indicators [16], and considering the representativeness of the indicators, the following three indicators are selected as conflict metrics.

Let *i* be an arbitrary time of the traffic conflict process between a right-turning motor vehicle and a non-motorized vehicle, and *i* ∊ [t_0_, min(T_c_, T_b_)]; where t_0_ represents the moment when its motion state begins to change due to the need for the object of the traffic conflict to take avoidance action; T_c_, T_b_ represent the time when the right-turning motor vehicle and the non-motorized vehicle are in the area of the traffic conflict, respectively. The meanings of other variables are shown in Table 3.

**Table 3:** List of variables.

Variable name/unit	Meaning
S_ci_, m	Traffic conflict distance of a right-turning motor vehicle at moment i
S_bi_, m	Traffic conflict distance of a non-motorized vehicle at moment i
V_ci_, m/s	Speed of a right-turning motor vehicle at moment i
V_bi_, m/s	Speed of a non-motorized motor vehicle at moment i
TTC_i_, s	Traffic conflict time at moment i
L, m	Length of a motorized vehicle
W, m	Width of a motorized vehicle
DST_i_, m/s^2^	Deceleration of a right-turning motor vehicle at Deceleration of right-turning motor vehicle at moment i
T_c1_, s	Time of right-turning motor vehicle entering traffic conflict area
T_c2_, s	Time of right-turning motor vehicle leaving traffic conflict area
T_b1_, s	Time of non-motorized vehicle entering traffic conflict area
T_b2_, s	Time of non-motorized vehicle leaving traffic conflict area

(1) **Traffic Conflict Time (TTC)**

TTC quantifies the time to collision during conflicts between right-turning motor vehicles and non-motorized vehicles. It is calculated as the minimum TTC value throughout the interaction. Two cases are analyzed: (1) the motor vehicle passes the conflict area first, and (2) the non-motorized vehicle passes first. The formula is:


TTC = min {TTCi} (1)

(2) **Deceleration Safety Time (DST**_**3**_)

DST assesses the deceleration required to avoid collisions, and DST_3_ is selected for this experiment to indicate the deceleration required for a right-turning vehicle to avoid colliding with a right-turning vehicle when the right-turning vehicle and pedestrians and non-motorized vehicles still have 3 seconds to meet. Right-turning vehicles’ trajectories and deceleration are calculated under two scenarios: (1) the motor vehicle exits the conflict area before non-motorized vehicles arrive, and (2) the non-motorized vehicle exits first. DST represents the minimum deceleration or maximum acceleration during the interaction.

(3) **Post-Encroachment Time (PET)**

PET measures the time difference between the first and second road users crossing the conflict zone, and different conflict levels are classified based mainly on PET in discrete choice modelling [17, 18].

It evaluates interaction safety and is calculated as

If the vehicle passes first:


$$\begin{array}{c} PET=T_{\frac{ped}{cyclist}}-T_{vehicle}\; \end{array}$$ (2)

If the pedestrian or cyclist passes first:


$$\begin{array}{c} PET=T_{vehicle}-T_{\frac{ped}{cyclist}} \end{array}$$ (3)

#### 2.2.2. K-Means.

This study employs the K-means clustering algorithm—a widely used unsupervised learning technique—to categorize human-vehicle conflict levels. The algorithm iteratively assigns data points to nearest clusters and updates centroids until convergence, offering computational efficiency for multidimensional data. While acknowledging its sensitivity to cluster geometry and the prerequisite to specify cluster count (K), we optimized clustering by testing multiple K-values through repeated executions.

Existing literature typically classifies conflicts into three levels (serious, general, minor) [4, 5]. To ensure comparability, we initially adopted K = 3. For comprehensive analysis, we additionally tested K = 2 (serious vs. general conflicts) to evaluate clustering effectiveness.

**Fig 1:**
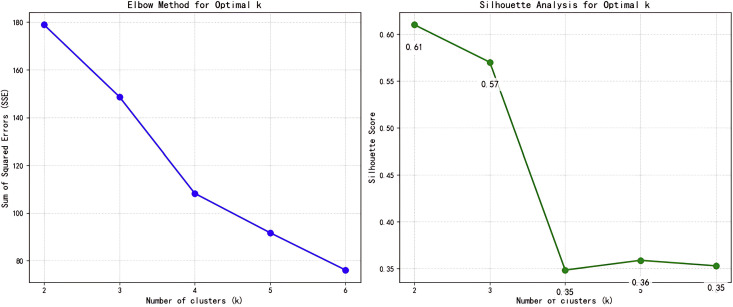
Results of Elbow and Silhouette Methods.

Quantitative validation confirmed K = 2 as optimal: Contour coefficient analysis yielded superior scores (K = 2: 0.61; K = 3: 0.57), indicating clearer separation with two clusters. The elbow method further supported this, showing significantly diminished SSE reduction beyond K = 2—confirming diminished returns from additional clusters. The result is shown in Fig 1.

#### 2.2.3. Regression models.

In this paper, factors affecting the severity of the conflict are analyzed using binomial Logit regression. Consider a vector with n independent variables, which means the dependent variable of the factors causing two different conflict severity levels is$\;x=(x_{1},x_{,}\ldots ,x_{n})$. Let the conditional probability *P(y = 1|x)* be the probability of occurrence of an event according to the observations relative to an event, then the Logit regression model is:


$$\begin{array}{c} P\left(y=\frac{1}{x}\right)=\frac{1}{1+e^{-g(x)}} \end{array}$$ (4)

Among them:


$$\begin{array}{c} g(x)=w_{0}+w_{1}x_{1}+\ldots +w_{n}x_{n} \end{array}$$ (5)

where *y* is the two values of conflict severity; $\;x_{i}$ is the independent variable that has a significant effect on conflict severity; $w_{0}$ is the constant; $\;w_{i}(i=1,,\ldots ,n)$ is the constant. Taking the logarithmic form of equation (18), it is obtained that:


$$\begin{array}{c} ln\frac{p}{1-p}=g(x)=w_{0}+w_{1}x_{1}+\ldots +w_{n}x_{n} \end{array}$$ (6)

To identify independent variables significantly affecting conflict severity (p ≤ 0.05), this study applied a 0.05 significance level throughout analysis. If at least one variable in the model has a statistically significant odds ratio (OR) with sig. < 0.05, the model is overall statistically significant. This study used the Hosmer-Lemeshow test to assess model fit. A p-value > 0.05 indicates good fit, meaning the current data’s information was appropriately captured. If the p-value meets this criterion, independent variables can be further discussed; otherwise, their statistical significance is considered poor.

## 3. Results

### 3.1. Severity of conflicts

#### 3.1.1. Clustering centers K = 3.

**Fig 2:**
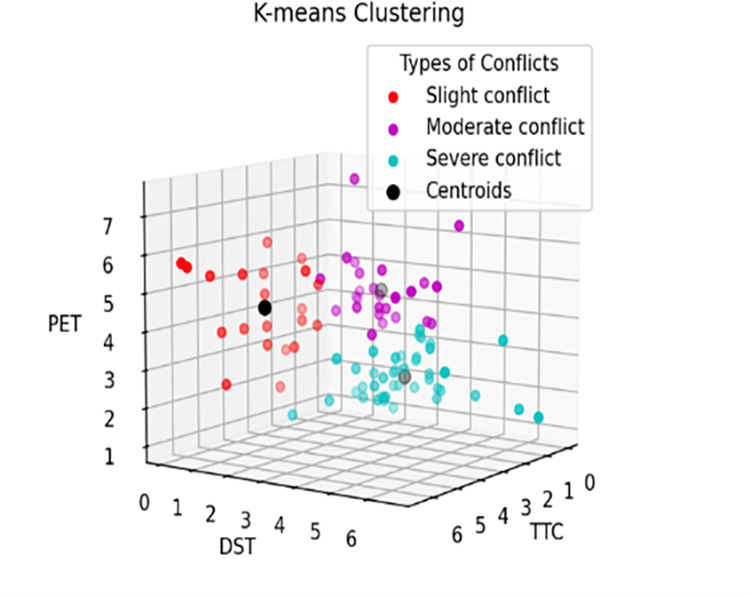
K = 3 Control Group.

**Fig 3:**
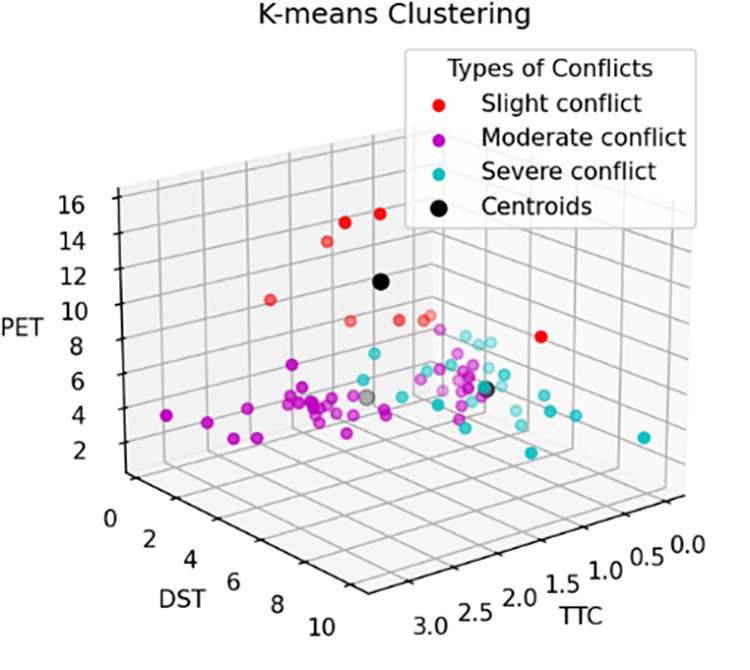
K = 3 Experimental Group.

**Fig 4:**
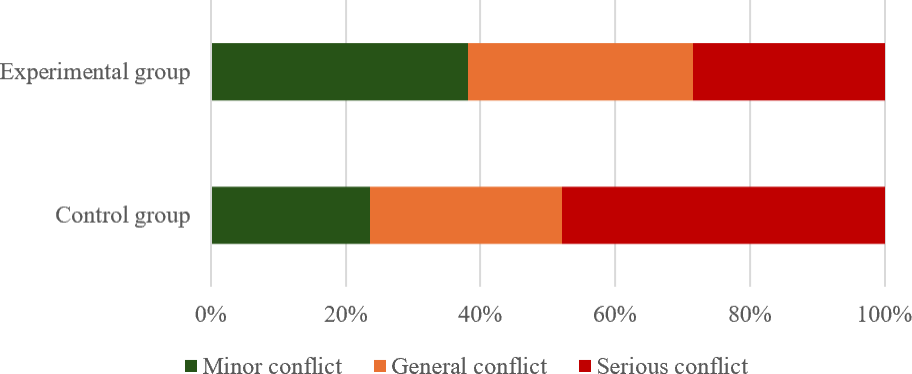
Different conflict severity at K = 3.

The reference thresholds for pedestrian-vehicle conflicts in the control group (Liyuan Road – Tianyuan Road and Suyuan Avenue – Qingshuiting Road) and the experimental group (Shuanglong Avenue – Qingshuiting Road and Suyuan Avenue – Chengxin Avenue) are shown in Table 4, Fig 2 and 3. In addition, as shown in Fig 4, the proportion of serious conflicts in the control group is the largest, accounting for 48% of the total, and the proportions of general and minor conflicts are close to each other; in the experimental group, minor conflicts account for 38%, which is the largest proportion, and serious conflicts only account for 28%, which is the smallest proportion.

**Table 4:** Clustering threshold (K = 3).

Control Group				Experimental Group			
Conflict indicators	TTC/s	DST_3_/(m/s^2^)	PET/s	Conflict indicators	TTC/s	DST_3_/(m/s^2^)	PET/s
Minor conflict	3.61	0.58	4.38	Minor conflict	1.67	1	2.43
General conflict	0.65	2.01	4.22	General conflict	0.59	2	4.94
Serious conflict	0.57	3.24	2.08	Serious conflict	0.3	3.06	2.36

#### 3.1.2. Clustering centers K = 2.

**Fig 5:**
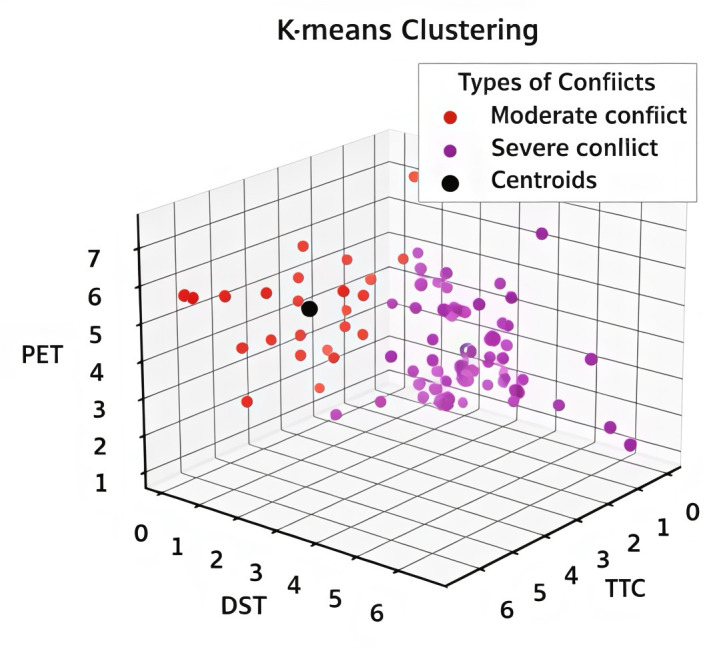
K = 2 Conrol Group.

**Fig 6:**
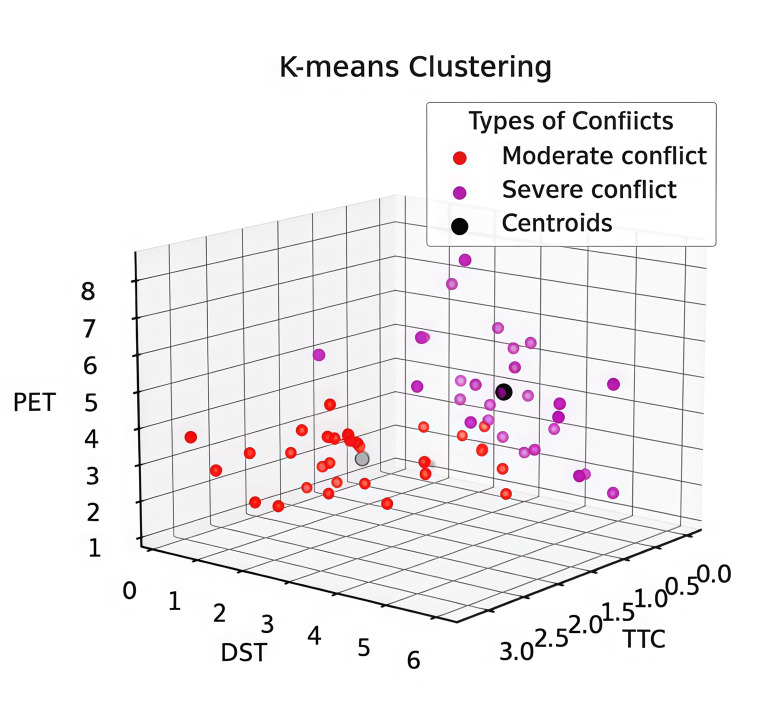
K = 2 Experimental Group.

**Fig 7:**
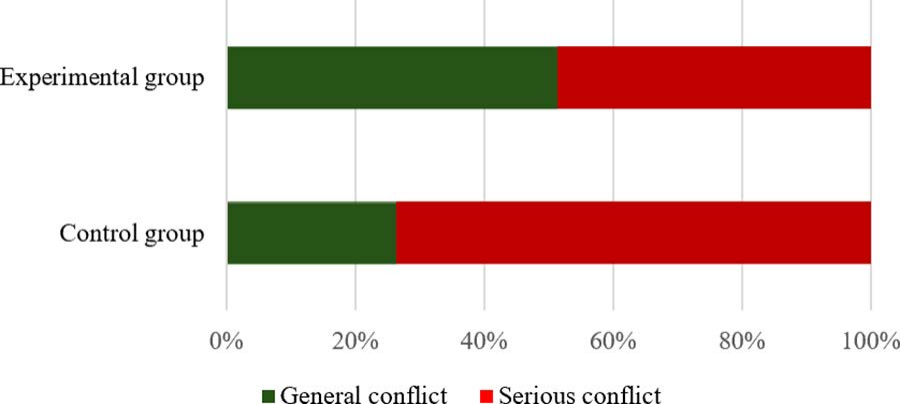
Different conflict severity at K = 2.

The reference thresholds of human-vehicle conflicts in the control and experimental groups are shown in Table 5, Fig 5 and 6. In addition, as can be seen in Fig 7, the number of severe conflicts is significantly lower in the control group, which accounts for 74%, and in the experimental group, which accounts for 49%.

**Table 5:** Clustering threshold (K = 2).

Control Group				Experimental Group			
Conflict indicators	TTC/s	DST_3_/(m/s^2^)	PET/s	Conflict indicators	TTC/s	DST_3_/(m/s^2^)	PET/s
General conflict	3.32	0.67	4.42	General conflict	1.34	1.36	2.34
Serious conflict	0.59	2.51	2.82	Serious conflict	0.48	3.2	2.12

Moreover, the selected conflict indicators PET, DST_3_, and TTC are empirically analyzed, and the specific values are calculated from each conflict event occurring between right-turning large vehicles and pedestrians and non-motorized vehicles, and the characteristics of the conflict indicators for the four study sites are shown in Table 6.

**Table 6:** Minimum, maximum, and average values of conflict indicators for each intersection.

Conflict indicators	Data type	A. Liyuan Road-Tianyuan Road	B. Suyuan Avenue-Qingshuiting Road	C. Shuanglong Avenue-Qingshuiting Road	D. Suyuan Avenue-Chengxin Avenue
PET(s)	Minimum	1.267	1.042	1.251	1.252
	Average	3.6038	3.01	3.145	2.718
	Maximum	8.75	6.257	7.023	5.32
DST_3_ (m/s^2^)	Minimum	0.308738	0.062	0.083	1.083
	Average	1.9937	2.024	1.903	3.011
	Maximum	3.361252	6.642	7.684	6.701
TTC(s)	Minimum	0.001976	0.004	0.0007	0.004
	Average	1.294	1.32	1.483	0.579
	Maximum	6.528465	6.32	8.406	2.613

**Fig 8:**
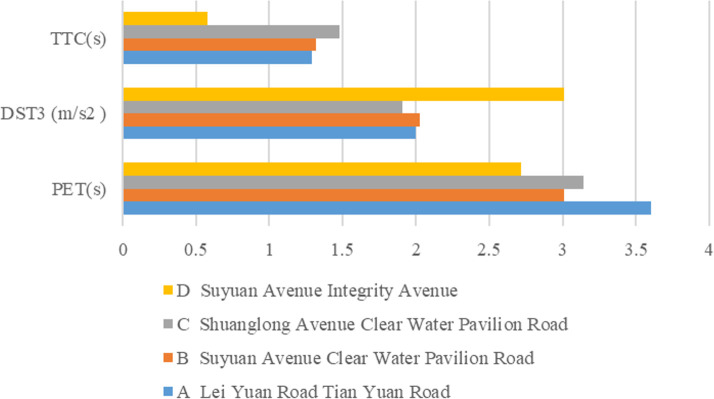
Mean values of conflict indicators by intersection.

Fig 8 shows that the average TTC value at the intersection of Liyuan Road – Tianyuan Road is higher, while the average DST_3_ value is lower, indicating that the conflict between motor vehicles and pedestrians and non-motorized vehicles and pedestrians at Liyuan Road – Tianyuan Road is minor because the location is close to a residential area, where most people drive, resulting in fewer cars and fewer people, and the conflict between motor vehicles and people is more minor; and the DST_3_ mean is the highest, while the average values of TTC and PET at the intersection of Suyuan Avenue – Chengxin Avenue are smaller. This suggests that there is more serious conflict between motor vehicles and pedestrians as well as non-motorized vehicles at the two intersections of Suyuan Avenue. This may be primarily because Suyuan Avenue is situated on a major traffic route, and the conflict is more severe because of the higher volume of pedestrians and traffic.

### 3.2. Influencing factors

The multivariate ordered Logit model, which categorized conflict severity into three levels, with a significance of P < 0.05 for one variable (see S1 Appendix for specific parameter estimates). It showed limitations due to small sample size, data quality biases, excessive categorization, low variable impact, and environmental specificity, making it statistically unreliable. These issues resulted in poor significance for most variables, reducing the model’s effectiveness. Consequently, the study adopted a binary Logit regression model, categorizing conflict severity into two levels. The Omnibus test of the model coefficients, the goodness in model fit, and the predicted probabilities yielded that the model was generally meaningful and the model fit was good (specific results are shown in S2 Appendix).

In this experiment, by observing Table 7, The significance of can be concluded that when P < 0.05, it means that a total of four factors, namely, whether there are motor-vehicle and non-motor-vehicle separation facilities on the entry lane in the environmental variable, speed at the stop line, whether or not to yield in the motor vehicle variable, and whether or not to cross the street within the zebra crossing in the pedestrian and non-motor vehicle variables, are related to the severity of the conflict. After selection by the backward: Logit regression, it can be concluded that other 14 variables have a significance of P > 0.05, and cannot be used to determine the severity of the conflict (specific calculations are shown in S3 Appendix).

**Table 7:** Variables in the equation.

	B	Standard error	Vardø (city in Finnmark, Norway)	(Number of) degrees of freedom (physics)	Significance	Exp(B)	95% Confidence interval for EXP(B)	
Lower limit	Limit
Non-isolated for imported machines	0.597	0.24	6.16	1	0.013	1.816	1.134	2.908
Yield	0.897	0.392	5.227	1	0.022	2.453	1.137	5.294
Crossing the street within the zebra crossing	−1.662	0.663	6.293	1	0.012	0.19	0.052	0.695
Speed-Stop Line	0.277	0.129	4.605	1	0.032	1.319	1.024	1.699
Constant	−2.752	0.784	12.313	1	0	0.064		

a. Variables entered at Step 1: Lane, Inlet non-segregated, Follow status, Merging traffic, Average speed, Exit non-segregated, Give way, Waiting time, Avoiding manoeuvre, Number of stops, Crossing in zebra crossing, Female, Child, Elderly, Type, Speed-stop line, Policy, Speed-20m.

From the coefficient B in Table 7, the import of no motor non-segregation, higher speeds and the behaviour of not giving way are positively correlated with the severity of the conflict, as indicated by the odd ratio (OR) of the lack of imported motor non-segregation and the increase in speed increase the chances of the severity of the conflict by about 81.6% and 31.9%, respectively. In addition, the behaviour of crossing the street within the zebra crossing was negatively associated with the severity of conflict, as implied by the OR (0.190) that the behaviour of crossing the street within the zebra crossing reduced the odds of conflict severity by about 81.0%.

The Logit regression model analysis reveals that the implementation of Stopping for Large Right-turning Vehicles policy exhibits limited statistical significance in mitigating conflict severity, indicating minimal efficacy in reducing serious traffic conflicts as intended. Instead, the regression coefficients in Equation 7 highlight four critical determinants shaping conflict dynamics:


$$𝐋𝐨𝐠𝐢𝐭\;𝐏=-2.752+0.597\times {𝐱}_{2}+0.897\times {𝐲}_{5}-1.662\times {𝐳}_{5}+0.277\times {𝐲}_{2\;}$$ (7)

First, the absence of clear machine/non-motorized segregation **(*x***_***2***_) exacerbates traffic flow conflicts between motorized and non-motorized users. This infrastructural deficiency not only increases collision risks but also obstructs drivers’ sightlines, delaying their detection of vulnerable road users and heightening the likelihood of severe incidents. Second, elevated vehicle speeds at stop lines **(*y***_***2***_) amplify collision risks through dual mechanisms—greater kinetic energy transfer during impacts and reduced driver reaction windows for evasive maneuvers. Third, large vehicles’ failure to yield **(*y***_***5***_) creates ambiguous right-of-way hierarchies at intersections, compounded by low compliance with traffic rules, which systematically elevates conflict potential. Finally, pedestrians and non-motorized users crossing outside designated zebra crossings **(*z***_***5***_) reflect diminished safety awareness, fostering unpredictable interactions with motorized traffic. Collectively, these factors underscore the multidimensional nature of intersection safety challenges, where infrastructural design, speed management, behavioral compliance, and user education intersect to shape conflict outcomes.

## 4. Discussions

### 4.1. Classification of conflict severity

By comparing the results for different numbers of clustering centers, it is concluded that since there is neither a right-turn hazard indication zone nor a large vehicle right-turn must-stop capture at the intersections of Liyuan Road – Tianyuan Road and Suyuan Avenue – Qingshuiting Road, the right-turning large vehicles do not slow down and stop in advance, and the conflicts with pedestrians and non-motorized vehicles are more obvious. On the other hand, the intetsections of Shuanglong Avenue-Qingshuiting Road and Suyuan Avenue-Chengxin Avenue are equipped with isolation zones and danger indication zones, which effectively alleviate the irregularity of large vehicles’ right-turning, and mitigate the conflicts between pedestrians, non-motorized vehicles and right-turning large vehicles, so the two intersections in the experimental group under autogenous clustering show more general conflicts and fewer serious conflicts. This suggests that appropriate measures under the Stopping for Large Right-turning Vehicles Policy can successfully reduce the intensity of collisions between right-turning large vehicles, non-motorized vehicles and pedestrians, prevent traffic accidents, and improve the overall safety level of the intersection.

The severe conflict threshold of TTC is low, but there are reasonable grounds. Firstly, it is the scenario specificity, the research object is a large vehicle turning right at low speed, the average speed of its turning is usually lower than 10 km/h, the physical braking distance and the avoidance time need to be shortened dramatically at this low speed, the traditional threshold based on the car is no longer applicable; secondly, it is the dynamic characteristics of the conflict, the large vehicle turning right with the blind spot of the inner wheel difference, the pedestrians/non-motorised vehicles often appear suddenly at a very short distance. Secondly, the logic of indicator combination, the study does not rely on TTC alone, but combines DST₃ and PET to make a comprehensive assessment, under this multi-indicator framework, a single TTC threshold should be interpreted with caution, and its role may be to assist in identifying extreme cases. Its role may be to assist in identifying extreme cases; finally, the control group validation, comparing intersections without policy implementation, which have a 48% share of severe conflicts, suggests that this threshold can effectively distinguish the risk difference before and after policy intervention, with internal consistency.

In addition, by comparing the two classifications, it is found that the difference between the clustering centers divided into two categories is more obvious, with a greater range of serious conflicts. As shown in Fig 7, the proportion of severe conflicts is three times that of general conflicts, reflecting the significant characteristics of the two controlled intersections.

### 4.2. Further Analysis of Impact Factors

#### 4.2.1. Potential Interaction Effects.

The four key factors identified in Equation (7) (lack of machine/non-separation *x*_*2*_, stop line speed *y*_*2*_, vehicle failure to yield *y*_*5*_, and crossing outside zebra crossing *z*_*5*_) may amplify conflict risk through synergistic effects.

(1) Superimposed effect of lack of segregation and vehicle speed: When an intersection lacks physical segregation, non-motorised vehicles are prone to intrude into the motorway. At this point, if the vehicle passes the stop line at a higher speed, the driver’s reaction time window and braking distance will be doubly compressed. The lack of segregation will further exacerbate the risk of blind spots for high-speed vehicles, forming a vicious cycle of infrastructure deficiencies & behavioural risks.
(2) Interaction between yielding and crossing: The failure to yield (*y*₅, OR=2.45) combined with illegal crossings (*z*₅, OR=0.19) exponentially increased conflict probability—validated by 145% higher severity odds when both factors coexisted (p < 0.01). When both are present at the same time, the right-of-way rules are completely ineffective – drivers lose their risk-avoidance initiative due to failure to yield, and the illegal crossing of vulnerable road users increases behavioural unpredictability, leading to an exponential increase in the probability of conflict.
(3) Moderation of zebra crossing effects by vehicle speed: Crossing within zebra crossings (*z*₅, OR=0.19) reduced severity odds by 81%, but this protective effect diminished at speeds >15 km/h (*y₂*, OR=1.32) due to compressed braking distances. When a vehicle slows down at a stop line, drivers have sufficient time to recognise pedestrians within the zebra crossing. However, if a vehicle approaches at high speeds, insufficient braking distance may still result in a collision even if pedestrians are within the zebra crossing.

#### 4.2.2. Summary of causes.

The analysis of the Stopping for Large Right-turning Vehicles Policy’s limited efficacy reveals multiple interconnected challenges. Primarily, insufficient enforcement mechanisms—stemming from inadequate monitoring infrastructure or inconsistent regulatory oversight—undermine policy compliance. This may be compounded by potential behavioral factors, such as drivers might frequently disregard parking requirements due to entrenched habits or momentary negligence, which could reflect a gap between policy intent and practical adherence. Simultaneously, infrastructural deficiencies emerge as critical barriers: obscured or absent stop signage fails to provide clear guidance, while unoptimized traffic flow designs exacerbate congestion during peak hours when mandatory stops disrupt existing movement patterns. These operational limitations intersect with systemic knowledge gaps, where insufficient public education campaigns leave both drivers and pedestrians uninformed about the policy’s safety objectives, perpetuating non-compliant behaviors. The cumulative effect of these factors—spanning enforcement lapses, human factors, design flaws, and awareness deficits—creates a complex ecosystem where well-intentioned regulations struggle to translate into measurable safety improvements.

#### 4.2.3. Suggested improvements.

This study proposes a systematic optimisation scheme for mitigating traffic conflicts, aiming to achieve synergistic improvement of road safety and efficiency through multi-dimensional interventions. The core strategy focuses on infrastructure reconstruction, behavioural reinforcement and dynamic management:

At the infrastructure level, the primary measures include deploying physical separation facilities (e.g., guardrails, green belts) to achieve spatial separation of motorised and non-motorised traffic flows, and simplifying the approach and exit routes at intersections to reduce the number of conflicting nodes. Traffic signage systems should be upgraded with highly recognisable yield signs, speed bumps and smart flashing warning devices to enhance visual guidance at key points, and adaptive signal technology to enable real-time response to traffic flow.

In terms of enforcement and education, a round-the-clock surveillance network will be set up and penalties for non-compliance (e.g., failure to yield to pedestrians, lane changing) will be increased. At the same time, a multi-channel public education programme should be implemented – using new media platforms to disseminate safety knowledge, and designing mixed traffic scenario simulation training courses for professional drivers to enhance the rule compliance of the whole user group.

Traffic management emphasizes fine spatiotemporal resource allocation: signal timing is dynamically adjusted via video detection and AI algorithms; motorized and non-motorized vehicles are separated during peak hours; and lane functional division is optimized using conflict heatmaps.

To ensure the continued effectiveness of the measures, it is recommended to build a data-driven assessment mechanism: regularly collect traffic data from multiple sources (conflict frequency, traffic delays, violation rates), use machine learning models to identify the evolution of risk patterns, and form a closed management loop of ‘monitoring-assessment-iteration’. Through the in-depth integration of the above technologies and management innovations, the intensity of intersection conflicts can be systematically reduced, and the urban transport system can be driven to evolve in a safer, more efficient and more inclusive direction.

## 5. Conclusions

This study presents an innovative empirical evaluation of the Stopping for Large Right-turning Vehicles Policy, leveraging high-resolution conflict data to advance understanding of urban intersection safety. By systematically extracting trajectory and attribute data of conflicts between right-turning trucks, pedestrians, and non-motorized road users, this study provides a detailed dataset for analyzing conflict severity. Using the K-means clustering algorithm, this study introduces a data-driven framework to classify conflict severity into distinct levels, offering a structured approach to quantify risks in complex traffic environments.

The quasi-experimental analysis comparing policy-implemented and control intersections reveals that the policy alone did not significantly reduce conflict severity. Instead, Logit regression modeling quantified four critical factors: absent segregation increased severity odds by 81.6% (OR=1.82), stop-line speeds >15km/h by 31.9% (OR=1.32), failure to yield by 145% (OR=2.45), and illegal crossings by 81.0% (OR=0.19). This finding challenges the primacy of behavioral mandates, highlighting the need for integrated solutions that combine infrastructure design (e.g., physical separation) with driver compliance mechanisms.

The study’s originality lies in its use of unsupervised learning for conflict categorization and determinant inference for policy evaluation, providing a robust, data-driven methodology to assess transport interventions. By demonstrating that effective safety improvements require holistic strategies rather than isolated policies, we offer actionable insights for policymakers, including enhanced infrastructure, stricter enforcement, and targeted public education. This study not only deepens theoretical understanding of intersection dynamics but also establishes a scalable framework for evaluating traffic policies through rigorous empirical analysis, contributing to safer and more efficient urban mobility systems.

While this study provides empirical insights into conflict severity between right-turning large vehicles and vulnerable road users, several limitations offer avenues for future research. First, the analysis is based on data from only four intersections in Jiangning District, limiting generalizability. Expanding the sample to include more diverse intersections could enhance the robustness of findings. Second, the evaluation of Stopping for Large Right-turning Vehicles Policy focuses on aggregate effects rather than granular enforcement dynamics (e.g., police enforcement intensity, driver compliance habits), which merit deeper exploration to refine policy design.

Methodologically, incorporating additional contextual factors—such as weather conditions, temporal traffic patterns, and flow variations—could enrich the understanding of conflict determinants. Furthermore, leveraging emerging technologies like artificial intelligence and big data analytics holds promise for improving the accuracy and efficiency of conflict analysis, enabling real-time risk assessment and adaptive policy interventions. By addressing these gaps, future research can provide more nuanced guidance for urban traffic safety management, contributing to evidence-based policies that enhance both safety and mobility.

## Supporting information

S1 Appendix Model Election. (DOCX)

S2 Appendix Model Effectiveness Test. (DOCX)

S3 Appendix Model Election. (DOCX)
