# Supplementary material for: Assessment of the stopping for right-turning large vehicles policy in Nanjing: Effectiveness and determinants
Source: PLoS One. 2025 Aug 26;20(8):e0319115. doi: 10.1371/journal.pone.0319115 (PMC12380282; doi:10.1371/journal.pone.0319115)
Supplement: S1 Appendix — (DOCX) [file pone.0319115.s001.docx]

**Appendix 1 Model Election**

Table 1 Parameter Estimates

|  | | **Significance** |
| --- | --- | --- |
|  |  |  |
| **Placement** | Speed 20m | .242 |
|  | Speed stop line | .931 |
|  | Waiting time | .354 |
|  | Average speed | .708 |
|  | Number of stops | .091 |
|  | [Lane=0] | .099 |
|  | [Importer non-isolated=0] | .000 |
|  | **[Importer non-isolated=1]** | **.025** |
|  | [Exporter non-isolated = 0] | .737 |
|  | [Exporter non-isolated = 1] | .329 |
|  | [Policy=0] | .310 |
|  | [Follower state=0] | .131 |
|  | [Converging traffic=0] | .254 |
|  | [Converging traffic=1] | .615 |
|  | [Let's go = 0] | .076 |
|  | [Row yielding = 1] | .335 |
|  | [Type=0] | .227 |
|  | [Type=1] | .772 |
|  | [Children=0] | .852 |
|  | [FEMALE=0] | .162 |
|  | [Crossing in zebra crossing = 0] | .144 |
|  | [Evasive action=0] | .427 |
|  | [Avoidance manoeuvre = 1] | .287 |
