# Supplementary material for: Assessment of the stopping for right-turning large vehicles policy in Nanjing: Effectiveness and determinants
Source: PLoS One. 2025 Aug 26;20(8):e0319115. doi: 10.1371/journal.pone.0319115 (PMC12380282; doi:10.1371/journal.pone.0319115)
Supplement: S2 Appendix — (DOCX) [file pone.0319115.s002.docx]

**Appendix 2 Model Effectiveness Test**

Table 1 Omnibus test for model coefficients

|  | | Chi-square (math.) | (Number of) degrees of freedom (physics) | Significance |
| --- | --- | --- | --- | --- |
| Step 15^a^ | Move | -1.572 | 1 | .210 |
|  | Lump (of earth) | 18.479 | 4 | .001 |
|  | Modelling | 18.479 | 4 | .001 |
| 1. Negative chi-square values indicate a decrease in chi-square value relative to the previous step. | | | | |

Table 2 Summary of Models

| Move | -2 log-likelihood | Cox Snell R-Square | Negolko R-square |
| --- | --- | --- | --- |
| 15 | 141.923^b^ | .136 | .189 |

*a. Estimation terminates at the 20th iteration because the maximum number of iterations has been reached. No final solution can be found.*

*b. The estimation was terminated at the fifth iteration because the parameter estimates varied by less than .001.*

Table 3 Classification table

|  | On-the-spot survey | | Anticipate | | | | | |
| --- | --- | --- | --- | --- | --- | --- | --- | --- |
|  |  |  | y | | | Percentage correct | | |
|  |  |  | 0 | 1 |  | | |  |
| Step 15 | y | 0 | 74 | 10 | 88.1 | | | |
|  |  | 1 | 28 | 14 | 33.3 | | |  |
|  | Overall percentage | |  |  | | | 69.8 | |
