# Supplementary material for: Assessment of the stopping for right-turning large vehicles policy in Nanjing: Effectiveness and determinants
Source: PLoS One. 2025 Aug 26;20(8):e0319115. doi: 10.1371/journal.pone.0319115 (PMC12380282; doi:10.1371/journal.pone.0319115)
Supplement: S3 Appendix — (DOCX) [file pone.0319115.s003.docx]

**Appendix 3 Model Election**

Table 1 Variables Not Included in Equation (7)

|  | | | Score | | (Number of) degrees of freedom (physics) | Significance |
| --- | --- | --- | --- | --- | --- | --- |
| Step 15^a^ | Variant | Carriageway | .799 | | 1 | .371 |
|  |  | Racing state | .472 | | 1 | .492 |
|  |  | Merge into traffic | .102 | | 1 | .750 |
|  |  | Average speed | 1.422 | | 1 | .233 |
|  |  | Non-isolated exporter | .850 | | 1 | .356 |
|  |  | Waiting time | .115 | | 1 | .734 |
|  |  | Avoiding action | .106 | | 1 | .745 |
|  |  | Number of stops | .007 | | 1 | .933 |
|  |  | Females | .689 | | 1 | .407 |
|  |  | Child | .195 | | 1 | .659 |
|  |  | Old man(or woman) | .984 | | 1 | .321 |
|  |  | Typology | .289 | | 1 | .591 |
|  |  | Formulation | .014 | | 1 | .905 |
|  |  | Speed - 20m | .053 | | 1 | .818 |
|  | Overall statistics | | | 5.855 | 14 | .970 |
